# Delayed Presentation of Pediatric Chronic Osteomyelitis of the Radius Following Cellulitis and a Prolonged Asymptomatic Period: A Case Report

**DOI:** 10.7759/cureus.113005

**Published:** 2026-07-20

**Authors:** Eriko Tsurunaga, Hideki Matsumura, Atsushi Yokota, Akira Ashida

**Affiliations:** 1 Department of Pediatrics, Saiseikai Suita Hospital, Suita, JPN; 2 Department of Pediatrics, Osaka Medical and Pharmaceutical University, Takatsuki, JPN; 3 Department of Orthopedic Surgery, Osaka Medical and Pharmaceutical University, Takatsuki, JPN

**Keywords:** cellulitis, chronic osteomyelitis, pediatric, prolonged asymptomatic period, radius, small colony variant

## Abstract

Pediatric cases of chronic osteomyelitis that remain asymptomatic for a prolonged period before becoming clinically evident are exceedingly rare. We report a rare case of pediatric chronic osteomyelitis of the left radius, which became clinically evident 1.5 years after an episode of left forearm cellulitis at the same anatomical site in a three-year-nine-month-old girl.

The patient presented with fever, left wrist pain, and difficulty in flexing her left fingers. Plain radiography and magnetic resonance imaging (MRI) revealed osteolytic lesions spanning the distal radial metaphysis and epiphyseal nucleus across the epiphyseal line, consistent with a chronic disease course. She underwent emergency surgical debridement and lavage of the radius. Blood culture results revealed methicillin-susceptible *Staphylococcus aureus*, and antibiotic therapy was adjusted accordingly. Tuberculous osteomyelitis was excluded. The patient completed a six-week course of antibiotic therapy and remained recurrence-free, with no evidence of growth disturbance, during follow-up.

The cellulitis resolved promptly with a one-week course of oral antibiotics, and the patient remained asymptomatic in the interim. This case underscores the importance of obtaining a careful history of prior skin infections at the same site when osteomyelitis is suspected, even when the infection appears to have resolved completely, and highlights the value of early MRI to facilitate prompt diagnosis given the potentially prolonged asymptomatic interval.

## Introduction

Osteomyelitis is classified as acute, subacute, or chronic based on its clinical course; however, the definition of chronic osteomyelitis varies considerably in the literature, with published case definitions requiring symptom duration ranging from as little as 10 days to as long as six months [1]. Among the proposed definitions, the most comprehensive requires symptoms suggestive of osteomyelitis that persist for ≥28 days, or a documented history of acute osteomyelitis accompanied by subsequent worsening symptoms, radiographic evidence of sequestrum or permeative lucencies, or readmission for osteomyelitis, and was adopted as the reference definition in this report [2]. In children, osteomyelitis typically follows an acute hematogenous course, and most cases are identified and treated promptly [3]. However, when inflammation is contained within the medullary cavity with minimal local signs or when a young child cannot clearly communicate symptoms, diagnosis may be delayed, leading to subacute or chronic osteomyelitis. Pediatric cases of chronic osteomyelitis that remain asymptomatic for a prolonged period before becoming clinically evident are exceedingly rare.

Epidemiologically, a recent systematic review of 41 studies comprising nearly 1,900 pediatric chronic osteomyelitis cases worldwide found a male predominance (58%) and involvement of the tibia or femur in two-thirds of cases, with *Staphylococcus aureus* identified in approximately three-quarters of cases with a single pathogen [1]. In an earlier single-center series, the vertebrae and femur were the most commonly involved sites, a predisposing risk factor such as preceding trauma, surgery, or hardware was identified in approximately two-thirds of cases, and cultures remained negative in roughly one-third of cases. A male predominance was also observed, although this trend did not reach statistical significance [4]. Chronic osteomyelitis in children has been classified into five pathogenetic categories: postacute hematogenous, primary hematogenous, contiguous focus, hardware-associated, and post-traumatic chronic osteomyelitis [2]. Notably, most epidemiological studies have focused on hematogenous spread or predisposing structural risk factors as the predominant routes to chronic infection, whereas contiguous spread from an adjacent skin or soft-tissue source has received comparatively little attention, despite being a recognized, if less common, pathogenetic category [2,5]. A clinically silent interval is not unprecedented in pediatric bone infections: Brodie's abscess, a well-circumscribed, sclerotic-walled lesion of subacute osteomyelitis, has been described as remaining asymptomatic for two to six months while remaining confined within its surrounding bony rim [6]. However, the 18-month interval observed in the present case substantially exceeds this typical range and was not associated with the discrete, encapsulated lesion characteristic of Brodie's abscess, suggesting that a different containment mechanism may have been operative. When a contiguous skin or soft-tissue infection is incompletely eradicated, residual bacteria may persist within the bone in a clinically silent state, potentially progressing to chronic osteomyelitis after a markedly delayed interval. Because the index skin infection often resolves uneventfully with standard outpatient therapy, clinicians may not associate a subsequent, temporally distant presentation with the earlier episode, further contributing to diagnostic delay.

Here, we describe a three-year-nine-month-old girl who developed chronic osteomyelitis of the left radius 1.5 years after an episode of forearm cellulitis at an identical anatomical site. To our knowledge, such an extended asymptomatic interval between a precipitating skin infection and clinically evident osteomyelitis has rarely been documented in pediatric literature. By presenting a detailed account of the diagnostic process, clinical course, and long-term outcomes of this case, and placing it within the current understanding of *S. aureus* bone persistence, this report aims to increase clinical awareness of contiguous osteomyelitis as a potential delayed sequela of skin and soft tissue infection.

This case was previously presented as a meeting abstract at the 49th Annual Meeting of the Japanese Society for Pediatric Infectious Diseases, held in Kanazawa, Japan, in October 2017.

## Case presentation

Patient history

The patient was a three-year-nine-month-old girl presenting with fever, left wrist pain, and difficulty in flexing her left fingers. Her medical history was notable for left distal forearm cellulitis at two years and three months of age, which was treated with a one-week course of oral cefdinir and resolved promptly, with no subsequent symptoms. She had no history of trauma, skin disease, atopic dermatitis, or pet ownership. Her vaccination history included four doses each of the *Haemophilus influenzae* type b, 13-valent pneumococcal conjugate, and diphtheria-pertussis-tetanus-inactivated poliovirus vaccines; one dose each of the bacille Calmette-Guérin (BCG) and the Japanese encephalitis vaccine; and two doses of the measles-rubella combined vaccine.

Presenting complaint and clinical course before admission

Four days before admission, the patient developed left wrist pain without an identifiable cause and visited a local orthopedic clinic the next day, where conservative management was recommended. Two days before admission, she developed difficulty flexing her left fingers and a fever of 39°C at night. She was reevaluated at the same clinic but was again managed conservatively. As the fever and pain persisted, the patient was referred to our department for further evaluation.

Physical examination on admission

Her body temperature, blood pressure, and heart rate were 36.5°C, 110/80 mmHg, and 120 beats/minute, respectively. Localized swelling and tenderness were observed over the distal left radius without overlying erythema or warmth. No sinus tracts or cutaneous discharges were observed. Because of the patient’s young age and limited cooperation, the range of motion of the wrist could not be formally assessed; however, spontaneous flexion of the fingers was restricted. No joint deformity was observed, and there were no findings suggestive of peripheral vascular compromise. Sensory status could not be reliably assessed at admission because of the patient’s age.

Laboratory and microbiological findings

**Figure 1:**
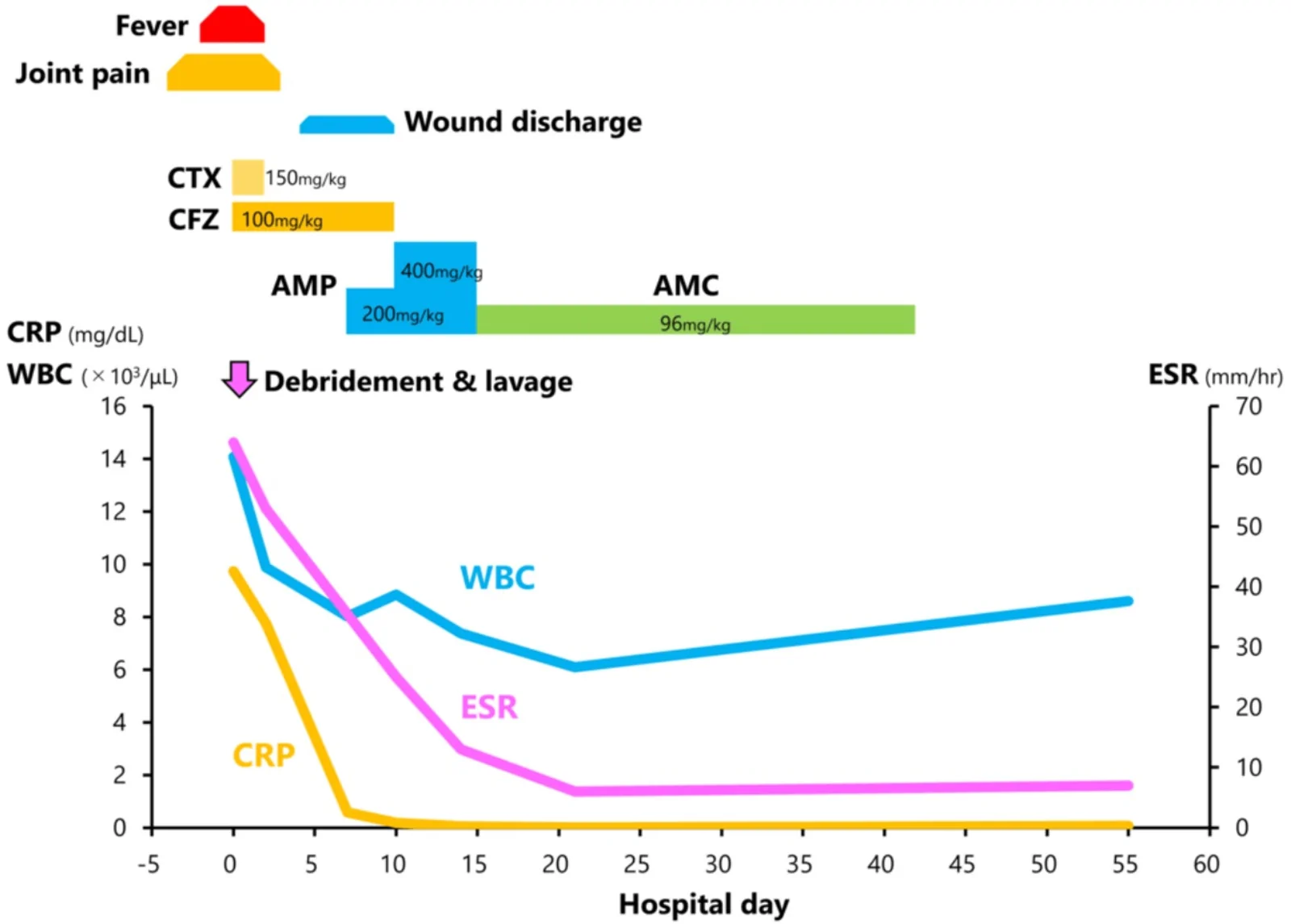
Patient’s clinical course after admission The time course of inflammatory markers (WBC, CRP, and ESR), fever, joint pain, and wound discharge is shown together with the antibiotic regimen CFZ: cefazolin; CTX: cefotaxime; AMP: ampicillin; AMC: amoxicillin-clavulanate; WBC: white blood cell count; CRP: C-reactive protein; ESR: erythrocyte sedimentation rate

Laboratory test results revealed leukocytosis with a left shift, elevated C-reactive protein and procalcitonin levels, and a markedly increased erythrocyte sedimentation rate, which were consistent with an active bacterial infection. Coagulation studies showed mild prolongation of the prothrombin time and prothrombin time international normalized ratio, likely reflecting an acute inflammatory state. Immunological workup, performed to exclude an underlying immunodeficiency given the unusual clinical course, showed elevated serum complement activity (total hemolytic complement), consistent with acute inflammation, while immunoglobulin fractions and neutrophil phagocytic and bactericidal functions were within normal limits, providing no evidence of immune dysfunction (Table 1). The microbiological findings are summarized in Tables 2, 3. The patient’s clinical course is described and illustrated in Figure 1.

**Table 1:** Laboratory findings on admission Reference ranges are for girls aged three years ^*^Results that are outside the reference range WBC: white blood cell; RBC: red blood cell; Hb: hemoglobin; Ht: hematocrit; Plt: platelets; TP: total protein; Alb: albumin; AST: aspartate aminotransferase; ALT: alanine aminotransferase; BUN: blood urea nitrogen; Cre: creatinine; CRP: C-reactive protein; PCT: procalcitonin; ESR: erythrocyte sedimentation rate; PT: prothrombin time; PT-INR: prothrombin time international normalized ratio; APTT: activated partial thromboplastin time; IgG: immunoglobulin G; IgA: immunoglobulin A; IgM: immunoglobulin M; CH50: total hemolytic complement

Parameter	Result	Reference range (3 year girl)
WBC (/μL)	14,080	5,500-15,500
Neutrophils (%)	55.0	32-72
Monocytes (%)	10.0	2-10
Lymphocytes (%)	35.0	25-65
RBC (×10⁶/μL)	4.83	3.9-5.3
Hb (g/dL)	12.4	10.8-14.0
Ht (%)	38.2	31-43
Plt (×10³/μL)	392	150-400
TP (g/dL)	7.7	6.0-7.7
Alb (g/dL)	4.0	3.5-4.2
AST (U/L)	33	24-44
ALT (U/L)	15	9-30
BUN (mg/dL)	9	6-20
Cre (mg/dL)	0.24	0.21-0.37
Na (mEq/L)	137	135-148
K (mEq/L)	4.7	3.6-4.8
CRP (mg/dL)	9.74^*^	<0.25
PCT (ng/mL)	0.20^*^	<0.05
ESR (mm/hour)	64^*^	5-15
PT (%)	60^*^	70-130
PT-INR	1.34^*^	0.87-1.03
APTT (seconds)	41.9^*^	28-36
D-dimer (μg/mL)	0.5	<1.0
IgG (mg/dL)	728	535-1,340
IgA (mg/dL)	82	24-167
IgM (mg/dL)	152	75-306
CH50 (U/mL)	84.1^*^	32-48
C3 (mg/dL)	162^*^	65-135
C4 (mg/dL)	36.9^*^	13-35
Phagocytic activity (E. coli) (%)	97.0	>88.0
Phagocytic activity (S. aureus) (%)	94.7	>92.0
Bactericidal activity (%)	93.0	83.2-94.8

**Table 2:** Microbiological findings on admission LAMP: loop-mediated isothermal amplification; BCG: bacille Calmette-Guérin; MSSA: methicillin-susceptible *Staphylococcus aureus*

Test	Result
Blood culture	*S. aureus* (MSSA)
Subcutaneous pus	*S. aureus* (MSSA) 1+
Bone marrow tissue	*S. aureus* (MSSA) 1+
Acid-fast bacilli culture	Negative
Tuberculosis LAMP assay	Negative
Tuberculin skin test	± (BCG-attributed)

**Table 3:** S. aureus antimicrobial susceptibility All agents tested: susceptible MIC: minimum inhibitory concentration

Agent	MIC (μg/mL)
Penicillin G	0.12
Oxacillin	≤0.25
Cefazolin	≤4
Cefmetazole	≤4
Imipenem	≤1
Amikacin	≤2
Gentamicin	≤0.5
Minocycline	≤0.5
Erythromycin	≤0.25
Clindamycin	≤0.25
Levofloxacin	0.25
Vancomycin	1
Teicoplanin	≤0.5
Linezolid	2
Fosfomycin	≤8
Trimethoprim-sulfamethoxazole	≤10

Imaging findings

**Figure 2:**
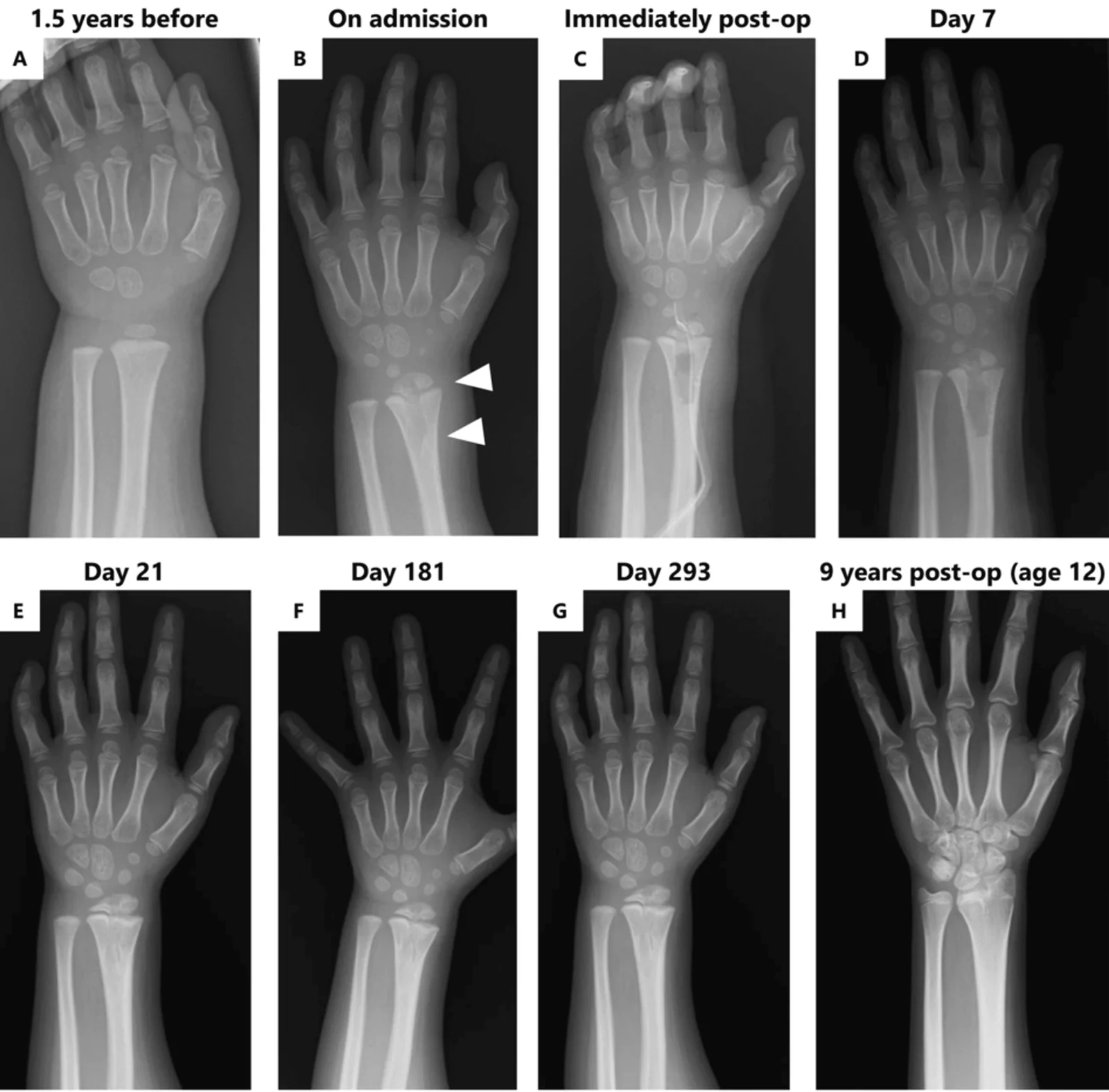
Serial plain radiographs of the left forearm Images are displayed with the distal end (i.e., fingertips) at the top. (A) 1.5 years before admission (at the time of the cellulitis episode): no bone abnormality. (B) On admission: soft-tissue swelling over the volar surface of the distal radius and osteolytic lesions spanning the distal radial metaphysis and epiphyseal nucleus across the epiphyseal line (arrowheads). (C) Immediately postoperatively. (D) Hospital day 7. (E) Hospital day 21. (F) Hospital day 181 after surgery. (G) Hospital day 293. (H) Nine years after surgery (age 12 years): partial closure of the radial epiphyseal line on the radial side without angular deformity

**Figure 3:**
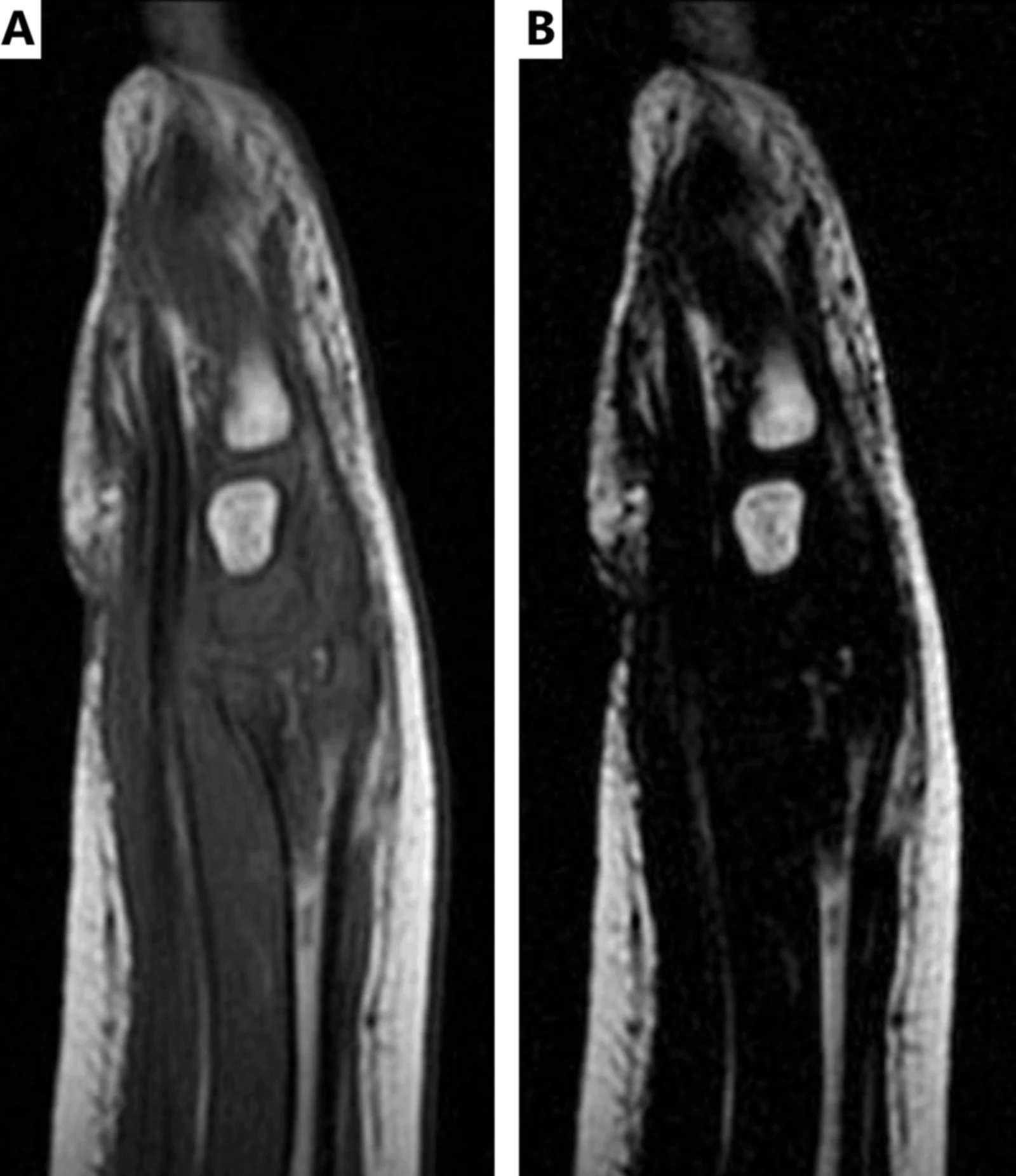
Magnetic resonance imaging of the left forearm at admission The images are displayed with the distal end (fingertips) at the top to match the orientation of the plain radiographs, as shown in Figure 2. (A) T1WI demonstrates a hypointense signal in the distal left radius. (B) Fat-sat T2WI of the same level shows heterogeneous high signal intensity, consistent with osteomyelitis. Edematous changes are also observed in the volar soft tissues of the wrist T1WI: T1-weighted image; Fat-sat T2WI: fat-suppressed T2-weighted image

Plain radiographs of the left forearm (Figures 2A-2H) revealed soft-tissue swelling over the volar surface of the distal radius and osteolytic lesions spanning the distal radial metaphysis and epiphyseal nucleus across the epiphyseal line (Figure 2B). Magnetic resonance imaging (MRI) of the left forearm (Figures 3A, 3B) revealed a hypointense signal on T1-weighted imaging and heterogeneous high signal intensity on fat-suppressed T2-weighted imaging at the same site, consistent with osteomyelitis, along with edematous changes in the volar soft tissues of the wrist.

Hospital course

On the basis of the MRI findings, we diagnosed the patient with left distal radial osteomyelitis with intra-articular extension, and emergency surgery was performed. On the day of admission, intravenous cefazolin (CFZ; 100 mg/kg/day) was initiated empirically to target *S. aureus*. Orthopedic surgeons performed debridement and lavage of the joint space and medullary cavity. Intraoperatively, a small perforation connecting the wrist joint to the medullary canal was identified, suggesting an intra-articular extension of inflammation and a prolonged chronic course.

Given this unexpected operative finding and the resulting open communication between the joint space and the medullary cavity, the empirical antibiotic regimen was broadened the following day by adding cefotaxime (150 mg/kg/day) to extend gram-negative coverage until the culture results became available. The patient’s condition improved the day after surgery, with progressive improvement in wrist pain and finger flexion postoperatively. No sensory disturbance was observed.

On the second day of hospitalization, results of blood cultures drawn at admission revealed methicillin-susceptible *S. aureus* (MSSA). Antibiotic therapy was simplified to CFZ monotherapy. To exclude tuberculous osteomyelitis, loop-mediated isothermal amplification assays were performed on intraoperative specimens from the radial fistula granulation tissue, medullary canal, and distal bone marrow, all of which yielded negative results. Histopathological examination of the intraoperative specimens revealed neutrophilic inflammatory infiltration of the wrist capsule, numerous bacteria along the bone fragments in the radial fistula, bone marrow granulation with neutrophilic infiltration in the radius, and neutrophil and lymphocyte infiltration of the cartilage. No histopathological features of tuberculosis were observed. The tuberculin skin test returned borderline positive (±) and was interpreted as a BCG vaccine-related response given her vaccination history. Therefore, tuberculous osteomyelitis was excluded from the differential diagnosis.

On hospital day 7, although inflammatory markers improved, fluid discharge was still observed from the distal wound margin. Antibiotic therapy was changed to ampicillin (AMP; 200 mg/kg/day), and the dose was subsequently increased to 400 mg/kg/day to optimize penetration into bone and soft tissues. The isolate was classified as susceptible to penicillin-class antibiotics based on the susceptibility panel, and favorable clinical and microbiological responses to AMP confirmed its in vivo activity.

On hospital day 14, inflammatory markers had normalized. Intravenous antibiotics were discontinued, and the patient was transitioned to oral amoxicillin-clavulanate (96 mg/kg/day). The patient was discharged on day 15 in a stable condition. The total duration of antibiotic treatment was six weeks (15 days of intravenous and 28 days of oral antibiotics).

The patient was regularly followed up at the Orthopedic Outpatient Department. Nine years after surgery, she was 12 years of age and was experiencing a pubertal growth spurt. Radiography showed partial closure of the radial epiphyseal line on the radial side but no angular deformity. To date, no recurrence of infection, growth disturbances, limb length discrepancies, or functional impairment has been identified.

## Discussion

Unusually prolonged asymptomatic course

The present case was characterized by an unusually prolonged asymptomatic interval before clinical manifestation, a feature that is exceedingly rare compared with typical pediatric osteomyelitis presentations [3]. Chronic osteomyelitis has been variably defined in the literature, with published case definitions in a recent systematic review requiring a symptom duration ranging from 10 days to six months, reflecting the absence of a single, universally accepted definition [1]. In the present patient, imaging at diagnosis revealed extensive deep-seated osteolytic lesions spanning the distal radial metaphysis and epiphyseal nucleus across the epiphyseal line, changes entirely inconsistent with acute osteomyelitis and indicative of a prolonged inflammatory process. Intraoperatively, a small perforation connecting the wrist joint to the medullary canal was identified, providing direct morphological evidence of intra-articular spread and chronic disease progression. These findings, combined with the 18-month asymptomatic interval, firmly established the diagnosis of chronic osteomyelitis. Consistent with the rarity of this presentation, an interval as long as 18 months, as observed in the present case, has rarely been documented [3,4].

Relationship between preceding cellulitis and osteomyelitis

The causative organism was confirmed as MSSA by blood culture, which is the most common pathogen in pediatric osteomyelitis [3]. Empirical therapy with CFZ was initiated upon admission, subsequently simplified to monotherapy upon MSSA identification, and later transitioned to AMP for enhanced bone and soft tissue penetration following the finding of prolonged wound discharge. Antimicrobial susceptibility testing of the intraoperative bone marrow curettage specimen demonstrated susceptibility to all agents tested, including penicillin G (minimum inhibitory concentration (MIC), 0.12 μg/mL), supporting the use of penicillin-based antibiotics. Although penicillinase production was not formally confirmed, the low penicillin G MIC and favorable clinical response to AMP supported the in vivo efficacy of penicillin-class antibiotics. Although the optimal duration of antibiotic therapy has not been firmly established, a recent systematic review found no clear relationship between the duration or route of antimicrobial therapy and rates of recurrence or orthopedic sequelae [1], consistent with a national survey documenting substantial variability in management practices among pediatric infectious disease physicians [7]. A total course of four to six weeks, following adequate debridement, is commonly recommended [8]. Notably, in a classic series of 163 children with osteomyelitis, those who received parenteral antibiotics for three weeks or less had a substantially higher rate of progression to chronic disease than those treated for longer than three weeks (19% vs. 2%) [9]. In the current case, thorough debridement and lavage were performed, inflammatory markers normalized prior to transition to oral therapy, and a total of six weeks of antibiotics were administered. The nine-year recurrence-free follow-up supports the adequacy of this treatment strategy.

A key clinical question in this case is the relationship between the initial cellulitis episode and subsequent osteomyelitis episodes. Notably, bacterial cultures were not obtained at the time of the cellulitis diagnosis. While this is clinically understandable, given the low yield in cellulitis [10], the absence of culture data prevented a direct microbiological comparison between the two episodes, thereby limiting the definitive confirmation of a causal association. Furthermore, cellulitis has a recurrence rate of 22%-49%, and subsequent episodes tend to be more severe [11], suggesting that an incompletely treated infection at this site could have contributed to its deeper spread into the bone. Therefore, coincidental hematogenous seeding cannot be ruled out. However, circumstantial evidence for a causal connection is compelling in this case. Osteomyelitis developed at the anatomically identical site as the preceding cellulitis; the patient had no history of trauma, new skin lesions, surgical procedures, or predisposing conditions, such as atopic dermatitis, that might suggest an alternative portal of entry. Furthermore, the initial cellulitis was treated with only a one-week course of oral antibiotics, a duration that, while appropriate for soft-tissue infection, would be insufficient to eradicate bacteria that had already colonized the adjacent medullary cavity. Supporting the plausibility of this scenario, a cohort study of pediatric patients hospitalized for cellulitis or abscesses of the distal extremities reported that contiguous osteomyelitis was detectable on imaging in nearly half of cases and that clinical presentation and laboratory findings alone could not differentiate patients with and without underlying bone involvement [5]. Taken together, we consider it most likely that cellulitis served as the precipitating event for osteomyelitis, with bacterial seeding of the radial medullary cavity occurring during the initial infection.

Proposed mechanism of occult persistence

Although *S. aureus* small colony variant (SCV) formation was not directly assessed in this patient and culture specimens were not specifically processed to detect this phenotype, we propose SCV-mediated dormancy as a plausible hypothesis-generating explanation for the prolonged asymptomatic interval. This hypothesis is supported by experimental evidence that SCVs constitute a quasi-dormant subpopulation capable of persisting within osteocytes, which lack efficient intracellular killing activity and may therefore serve as a niche for occult bacterial spread through the lacunocanalicular network [12-15]. Under this model, bacteria seeded into the medullary cavity at the time of the initial cellulitis episode could have entered a dormant, antibiotic-tolerant state, evading both host clearance and the short course of oral antibiotic therapy administered for the skin infection before eventually reactivating to produce the clinically evident osteomyelitis observed 18 months later. We emphasize that this mechanism remains unconfirmed in the present case and is proposed as a framework for future investigations rather than an established explanation.

Clinical implications

The current case provides important diagnostic insight. When osteomyelitis is suspected in children, a detailed history of prior skin and soft-tissue infections at the affected site should be obtained, as the interval between the precipitating infection and the manifestation of osteomyelitis may be prolonged. Early MRI is recommended because plain radiographs may not reveal abnormalities in the early stages of osteomyelitis [3]. When cellulitis occurs in the distal extremities of children, clinicians should maintain a low threshold for imaging to exclude contiguous osteomyelitis [5]. Additionally, obtaining bacterial cultures at the time of cellulitis presentation, while rarely productive, should be considered when the clinical context raises concern for deeper infection, as it would allow microbiological comparison if osteomyelitis subsequently develops.

Finally, the inflammatory process in this case extended beyond the epiphyseal line, raising substantial concerns about potential growth disturbances, including limb-length discrepancies or angular deformities. Although no such complications, functional impairment, or recurrence of infection have been identified at nine years after surgery, the patient is now 12 years of age and undergoing her pubertal growth spurt, a period of rapid skeletal growth during which latent growth disturbances may become apparent. This highlights the limitations of long-term outcomes and underscores the need for continued orthopedic surveillance.

Limitations

This report has several limitations. As this was a single case, its findings may not be generalizable. Bacterial cultures were not obtained at the time of the initial cellulitis, limiting the confirmation of a causal link with subsequent osteomyelitis. The proposed SCV-mediated mechanism has not been directly assessed and remains hypothetical. Formal assessment of wrist range of motion and sensory status was not possible because of the patient’s age. As the patient is currently undergoing a pubertal growth spurt, continued orthopedic surveillance is warranted to detect any latent growth disturbances.

## Conclusions

This case highlights that osteomyelitis can develop as a delayed complication of preceding cellulitis, with a clinically silent interval that may extend well beyond the timeframe typically associated with bone infections. Clinicians evaluating a child with suspected osteomyelitis should specifically elicit a history of prior skin or soft-tissue infections at the same anatomical site, even when the infection appears to have been resolved completely months or years earlier. When such a history is present or when clinical findings raise concerns for occult bone involvement, early MRI should be pursued to enable prompt diagnosis and timely surgical intervention.
